# Concurrent adult pulmonary tuberculosis prevalence survey using digital radiography and Xpert MTB/RIF Ultra and child interferon-gamma release assay *Mycobacterium tuberculosis* infection survey in Karachi, Pakistan: a study protocol

**DOI:** 10.12688/wellcomeopenres.15963.1

**Published:** 2020-07-06

**Authors:** Palwasha Y. Khan, M. Shariq Paracha, Chris Grundy, Saadia Saeed, Maqboola Dojki, Falak Madhani, Liesl Page-Shipp, Nazia Khursheed, Waleed Rabbani, Najam Riaz, Saira Khowaja, Owais Hussain, Ali Habib, Uzma Khan, Katharina Kranzer, Rashida A. Ferrand, James J. Lewis, Aamir J. Khan, Katherine L. Fielding

**Affiliations:** 1 Interactive Research and Development, Karachi, Pakistan; 2 Department of Infectious Disease Epidemiology, London School of Hygiene & Tropical Medicine, London, UK; 3 TB Centre, London School of Hygiene & Tropical Medicine, London, UK; 4 The Indus Health Network, Karachi, Pakistan; 5 Interactive Research and Development Global, Singapore, Singapore; 6 Interactive Health Solutions, Karachi, Pakistan; 7 Interactive Research and Development Global, Dubai, United Arab Emirates; 8 Clinical Research Department, London School of Hygiene & Tropical Medicine, London, UK; 9 Y Lab – the Public Services Innovation Lab for Wales, Cardiff University, Cardiff, UK

**Keywords:** M. tuberculosis transmission, tuberculosis prevalence, infectious tuberculosis, Karachi

## Abstract

**Background: **Assessment of the effectiveness of tuberculosis control strategies requires the periodic measurement of *M. tuberculosis *transmission in populations, which is notoriously difficult. One well-established method is to measure the prevalence of infectious pulmonary tuberculosis in the population which is then repeated at a second time point after a period of ‘intervention’, such as scale up of the Search-Treat-Prevent strategy of the Zero TB Cities initiative, allowing for a ‘before and after’ comparison.

**Protocol: **The concurrent adult pulmonary tuberculosis prevalence survey (using digital radiography and Xpert MTB/RIF Ultra) and child *M. tuberculosis *infection survey (using QuantiFERON-TB® Gold Plus) will primarily provide a baseline measure of the burden of adult infectious tuberculosis in Karachi and assess whether a large-scale interferon gamma release assay survey in children aged 2 to 4 years is feasible. The target population for the prevalence survey is comprised of a stratified random sample of all adults aged 15 years and above and all children aged 2 to 4 years resident in four districts in Karachi. The survey procedures and analyses to estimate pulmonary tuberculosis prevalence are based on the World Health Organization methodology for tuberculosis prevalence surveys.

**Ethics and dissemination: **The study protocol has been approved by the Interactive Research Development / The Indus Hospital Research Centre Research Ethics Committee in Karachi, Pakistan and the London School of Hygiene & Tropical Medicine Research Ethics Committee. Due to non-representative sampling in this setting, where a large proportion of the population are illiterate and are reluctant to provide fingerprints due to concerns about personal security, verbal informed consent will be obtained from each eligible participant or guardian. Results will be submitted to international peer-reviewed journals, presented at international conferences and shared with participating communities and with the Provincial and National TB programme.

## Introduction

Current strategies are failing to contain the global tuberculosis epidemic, and are far from achieving the sustained reductions in tuberculosis disease incidence of up to 17% per year which are required to meet the targets set out in the World Health Organization (WHO) End TB Strategy by 2035 ^1^. A paradigm shift in global tuberculosis control strategy is needed in order to break the *Mycobacterium tuberculosis* ( *M. tuberculosis*) ** transmission cycle and make real progress in ending the epidemic.

The Zero TB Cities Initiative is one such approach which endorses the implementation of integrated evidence-based interventions at the community-level. The aim of the initiative is to create “islands of tuberculosis elimination” through (i) active case-finding (ACF) using systematic and evidence-based diagnostic algorithms (‘search’), (ii) early and effective treatment (‘treat’) and (iii) a reduction in *M. tuberculosis* exposure through infection prevention and control in healthcare facilities including ultraviolet germicidal irradiation and provision of preventive therapy for high risk groups (‘prevent’) ^2^. This ‘Search-Treat-Prevent’ strategy has been adopted by key stakeholders including the Stop TB Partnership and a number of non-governmental organisations - Partners in Health, Advance Access & Delivery, Interactive Research and Development (IRD), underpinned by academic input from the Centre for Global Health Delivery-Dubai, Harvard Medical School, in a number of cities, namely Chennai, Lima and Karachi ^3^. However the effectiveness of this comprehensive ‘Search-Treat-Prevent’ approach in reducing *M. tuberculosis* transmission at a population-level in large, urban high tuberculosis burden settings in the 21st century is unclear.

Assessment of the effectiveness of such tuberculosis control strategies requires the periodic measurement of *M. tuberculosis* transmission in populations, which is notoriously difficult and relies on proxy measures, assumptions and combination approaches ^4^. One well-established method is to measure the prevalence of infectious pulmonary tuberculosis in the population, which provides an estimate of the burden of diagnosed and undiagnosed culture-confirmed tuberculosis in a population at a given time. The prevalence survey can then be repeated at a second time point after a period of ‘intervention’, such as scale up of the Search-Treat-Prevent strategy, allowing for a ‘before and after’ comparison. However, a reduction in the prevalence of adult pulmonary tuberculosis may occur because of several reasons, such as a reduction in *M. tuberculosis* transmission or in a reduction in progression from infection to disease or a reduction in disease duration or even because of secular trends, e.g. improvement in socio-economic conditions or mass in- or out-migration. In addition, in settings where the majority of current active disease is the result of reactivation from historic transmission, *M. tuberculosis* transmission may show a decrease with minimal reduction in prevalence. Interpretation of any changes in adult pulmonary disease prevalence will therefore be aided by simultaneously undertaking a survey which measures another parameter of *M. tuberculosis* transmission at a population-level, such as risk of *M. tuberculosis* infection ^5^. Accurately determining the prevalence of *M. tuberculosis* infection in a population allows derivation of an average annual risk of *M. tuberculosis* infection risk (ARTI), under the assumption of no change in risk over calendar time. ARTI therefore reflects cumulative lifetime infection risk and measures the force of infection with amount of delay determined by the age group in which the *M. tuberculosis* infection prevalence survey is conducted. Acknowledging the differences in transmission dynamics between adults and children, choosing younger age groups has the advantage of calculated ARTI being bracketed within a relatively narrow period between the average birth date of the group and the date of the survey ^6^. Interferon gamma release assays (IGRAs) are one way of inferring infection status and have a higher specificity compared to tuberculin skin tests (TST), especially in young recently BCG-vaccinated children ^7^. However, these diagnostic tests have not been implemented at scale in children aged under five years in high-burden resource-limited settings.

### Study rationale

The rationale for this concurrent adult pulmonary tuberculosis prevalence survey and child IGRA survey in Karachi is to primarily provide a baseline measure of the burden of infectious tuberculosis (culture-confirmed adult pulmonary tuberculosis) in 2018 to 2019 (at the start of fully scaled up implementation of the Zero TB Karachi initiative) and to see if a large-scale IGRA survey in children aged 2 to 4 years is feasible. An IGRA survey in children aged 2–4 years, if feasible would provide an estimate of the ARTI over the last three years. 

In brief, the Zero TB Karachi initiative involves ACF using mobile digital radiography interpreted by computer-aided detection of tuberculosis software, CAD4TB™ (Diagnostic Image Analysis Group, Radboud University Medical Center, Nijmegen, the Netherlands) and sputum examination using Xpert MTB/RIF (Cepheid, Sunnyvale, CA, USA); post-exposure treatment of household contacts of index patients with drug-sensitive tuberculosis; ultraviolet germicidal irradiation (UVGI) light installation in high-volume outpatient departments and pulmonary medicine wards; strengthening of screening and management of childhood tuberculosis; and integrated case-based data capture using an Android application linked to the OpenMRS platform across the whole of the city.

The aim is to assess whether a reduction in person-to-person *M. tuberculosis* transmission has occurred as result of the Zero TB interventions in Karachi. The adult tuberculosis prevalence and child IGRA survey described here will serve as the baseline measure and will be repeated in four years’ time (an end line survey in 2022–2023) using the same methodology as described here with the aim to conduct a before-and-after comparison.

### Study objectives

The primary objective of this study is to estimate the period prevalence of culture-confirmed (diagnosed and undiagnosed) adult pulmonary tuberculosis in Karachi in 2018 to 2019.

The secondary objectives are to assess the feasibility of conducting a large-scale IGRA survey in young children and estimate the ARTI in children aged 2 to 4 years in Karachi. If the child IGRA survey is feasible then the plan would be to repeat the IGRA survey as part of the end line survey in 2022–2023 in children aged 2 to 8 years stratified by those born after the implementation of Zero TB (children aged 2 to 4 years) and those born before/during early implementation of the Zero TB initiative (children aged 5 to 8 years).

### Study outcomes and process measures

1. *Adult tuberculosis prevalence survey:*
  Primary outcome
  i. Prevalence of all culture-confirmed pulmonary tuberculosis including those individuals already diagnosed and on treatment
2. *Child M. tuberculosis* infection survey and *IGRA feasibility study*:
  Secondary outcomes
  i. Feasibility-related:
    a. proportion of eligible children who were enrolled of total eligible population (participation)
    b. proportion of valid IGRA results obtained
  ii. ARTI based on the prevalence of *M. tuberculosis* infection in children aged 2–4 years

***Process measures***

1. *Adult pulmonary tuberculosis prevalence survey:*
  i. Proportion of eligible participants screened with symptom screen and chest x-ray (CXR)
  ii. Proportion of participants eligible for sputum collection who submitted sputum
  iii. Proportion of sputum samples contaminated
2. *M. tuberculosis infection prevalence survey in young children and IGRA feasibility study:*
  - Proportion of eligible participants with successful venepuncture
  - Proportion of samples in the laboratory within 16 hours of blood draw
  - Proportion of indeterminate IGRA tests

## Methods

### Study design

A cross-sectional study of infectious (bacteriologically confirmed diagnosed and undiagnosed) adult pulmonary tuberculosis using digital radiography and Xpert MTB/RIF Ultra (Cepheid, Sunnyvale, CA, USA) and a cross-sectional study of *M. tuberculosis* infection using QuantiFERON-TB® Gold Plus (Qiagen, Germantown, USA) in young children will be undertaken in Karachi, the provincial capital of Sindh province. 

### Study period and setting

Recruitment began in March 2018 and will continue until the target sample size has been achieved. 

Due to logistical constraints, namely travel time and limited resources, the survey will be restricted to four districts of Karachi (Korangi, Karachi Central, Karachi South and parts of Karachi West). These districts include approximately 65% of the total 14.9 million population of Karachi as per the 2017 census ^8^. All included districts are each made up of a combination of formal and informal settlements (slums) known locally as *katchi abadis.* The survey covers both the formal and informal settlements to ensure a representative sample. Several cantonment areas which serve as military, air force and naval bases with associated residential areas are excluded as access to these areas are restricted and require special clearance to enter.

**Figure 1.**
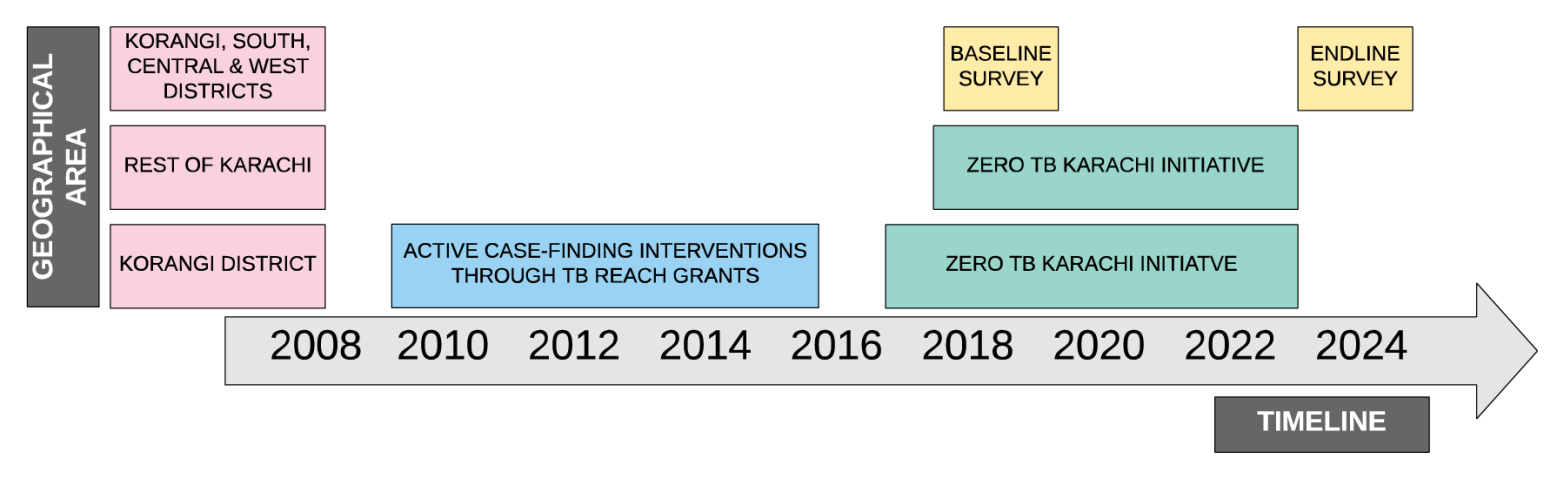
Timeline of tuberculosis control interventions by geographical area.

Prior to the Zero TB Karachi initiative, the Indus Hospital, a free-of-charge healthcare facility located in Korangi district, in collaboration with Interactive Research and Development (IRD), a not-for-profit organisation, received several grants to increase tuberculosis patient notifications from the private sector over the period of 2010–2015 as part of the TB REACH initiative of the Stop TB Partnership and the Unitaid-funded TBXpert project. Korangi district was the designated focus for implementation of these TB grants as the population in this area was identified as one of the most disadvantaged in Karachi with poor access to healthcare. The first of these grants funded an extensive ACF campaign in 2011 in Korangi district, which resulted in a doubling of tuberculosis notification rates in this part of Karachi compared to an adjacent control area ^9^. Varying intensity of ACF has continued in this district since 2011 and the resulting infrastructure and social community mobilisation from these grants, including the Xpert MTB/RIF scale-up in the city in 2013–2014 continues to impact on tuberculosis case-finding. Figure 1 below illustrates the timeline of the implementation of tuberculosis control interventions from 2008 to 2024.

### Participants

The target population for the prevalence survey is comprised of a stratified random sample of all adults (males and females including pregnant women) aged 15 years and above, resident in the four districts, and the target population for the IGRA survey includes a stratified random sample of all resident children aged 2 to 4 years in the same four districts.

*Inclusion criteria:* Household members, defined as those who have slept in the household the night before the start of enumeration in the selected neighbourhood block are eligible for inclusion in the survey.

*Exclusion criteria:* Individuals who are resident in public and private institutions which require special clearance (prison, military/air force/naval, diplomatic compounds, schools, hotels, refugee camps and health facilities) are excluded.

### Study sample size calculation

***Adult pulmonary tuberculosis prevalence survey.*** A nationwide cross-sectional prevalence survey of adult pulmonary tuberculosis was conducted in Pakistan in 2010–2011 which found prevalence estimates for smear and culture confirmed tuberculosis of 282 per 100,000 population ((95% confidence interval (CI) 161–402 per 100,000) and 363 per 100,000 population (95% CI 229 - 497 per 100,000), respectively), in Sindh province ^10^. Assuming a higher prevalence of culture confirmed tuberculosis in the megacity of Karachi compared to the provincial estimate, an estimate of 400 per 100,000 population of culture confirmed pulmonary TB cases was used for the sample size calculation. Thus a target sample of 21,668 enumerated adults will provide a relative precision of 25% assuming a design effect of 1.128 (D=1 + *m*k ^2^µ ^11^, where *m*= 200 [number of individuals per cluster], k=0.4 [coefficient of variation] and µ=0.004 [prevalence estimate]), and an expected participation rate of 75%.

However, to detect a reduction in tuberculosis prevalence in a follow-up survey ideally to take place in 4–5 years’ time and assess whether Zero TB Karachi initiative has had an impact on transmission, the sample size will have to be substantially larger. To have at least 80% power to detect a 33% reduction in tuberculosis prevalence in the follow-up survey (compared to the baseline survey) with a minimum participation rate of 75% in each survey, a sample of at least 43,734 at baseline and a similar sample size in 4 years’ time would be required. Therefore, our overall target sample size for this survey is 44,000.

***M. tuberculosis infection prevalence survey in young children (2- to 4-year-olds).*** We did not conduct a formal sample size calculation for the child *M. tuberculosis* infection prevalence survey as the primary objective is to assess feasibility of undertaking a large-scale community IGRA survey in this population. 

### Sampling method

*Rationale for sampling design with oversampling of Korangi district:* We hypothesise that the prevalence of adult culture-confirmed pulmonary tuberculosis may be lower in Korangi district, despite being one of the most poverty-stricken districts, due to the longer-term ACF efforts implemented in the district (see Figure 1). Although the aim of this study is not to assess a difference in the burden of disease across different parts of Karachi, it is important to quantify (albeit crudely) any differences in the prevalence of adult tuberculosis and *M. tuberculosis* infection in children at baseline of the Zero TB Karachi Initiative across districts. Without these data, interpretation of findings from the follow-up survey assessing the impact of the Zero TB Karachi Initiative in 4–5 years’ time will be difficult.

*Sampling approach*: A cluster sampling method for the survey has been employed, with the *a priori* plan to oversample Korangi district due to the potentially lower prevalence of adult culture-confirmed pulmonary tuberculosis (see above). For ease of description of the sampling method, we define Korangi district as the ‘prior ACF’ zone and Karachi South, Central and West districts as the ‘no prior ACF’ zone. A total of 44,000 adults will be invited to participate in the study stratified by zone: 22,000 individuals from Korangi district (‘prior ACF’ zone) and 22,000 individuals from the ‘no prior ACF’ zone (Karachi South, Karachi Central and Karachi West districts). This same stratified sampling strategy by zone will be employed in the end line survey in 2022–2023. Table 1 shows how the study population is stratified by district and the number of neighbourhood blocks to be sampled per district.

**Table 1.** Target screening population and number of neighbourhood blocks to be surveyed stratified by prior ACF zone and No prior ACF areas.

	Prior ACF zone		No prior ACF zone	
	Korangi district	Karachi South district	Karachi Central district	Karachi West district
Adult population (million) ^1^	1.7	1.3	2.1	2.7
Percentage of adult population residing in the *katchi abadis* at district level ^2^	8	10	18	8
Number of *tehsil* selected / total number of tehsil per district (%)	4/4 (100)	4/7 (57) *	5/5 (100)	4/8 (50) **
Target adult population for TB prevalence survey screening	22,000	6,000	9,500	6,500
Percentage of total adult population to be sampled per district (%)	1.3	0.5	0.5	0.2
Total number of neighbourhood blocks to be surveyed ^3^	110	30	48	33
Number of neighbourhood blocks defined as *katchi abadis* to be sampled	9	3	9	3

1 Estimated adult population calculated using total population of district as per Pakistan Bureau of Statistics 2017 census data multiplied by 0.7 based on demographic structure of Pakistan that 70% of population is ≥15 years (Data sources: Pakistan Bureau of Statistics http://www.pbs.gov.pk/content/block-wise-provisional-summary-results-6th-population-housing-census-2017-january-03-2018 and CIA.gov: https://www.cia.gov/library/publications/resources/the-world-factbook/geos/pk.html)

2 Data provided by the Sindh Katchi Abadi Authority (unpublished)

3 Assuming an average of 200 adults per block

* Several *tehsils* in Karachi South were found to be highly commercial areas and inaccessible to mobile vans during a pilot run of operations in 2017 so were excluded from the sampling frame

** Includes only those *tehsils* of Karachi West which border Karachi Central and Karachi South for logistical reasons (transport time of mobile x-ray vans from headquarters of ≤ 2 hours)

*katchi abadis* informal settlements *; tehsils* administrative unit of a district

Each district is made up of administrative units called ‘ *tehsils’* (subdivisions). We calculated the number of neighbourhood blocks per *tehsil* to be sampled in each district based on the adult population size of each *tehsil* as per the 2017 Census of Pakistan ^8^. Some of the *tehsils* in Karachi South are predominantly commercial areas and are inaccessible to mobile x-ray vans, as found during a pilot run in 2017 and so have been excluded from the sampling frame. In addition, we will only sample those *tehsils* in Karachi West which border Karachi Central and Karachi South for the logistical reason of keeping the transport time of the mobile x-ray vans to under 4 hours per day.

Data provided by the Sindh Katchi Abadi Authority have been used to estimate the proportion of the population residing in *katchi abadi* (informal settlements) within each district (ranging from 8 to 18%). These data will guide the selection of neighbourhood blocks from *katchi abadis* to be consistent with the overall proportion of the population residing in *katchi abadis* at the district level. For example, of the 110 neighbourhood blocks in Korangi district, 9 neighbourhood blocks will be selected from areas defined as *katchi abadis*, and the remaining 101 neighbourhood blocks will be selected from other areas to reflect the 8% of the population of that district which live in *katchi abadis*. 

**Figure 2.**
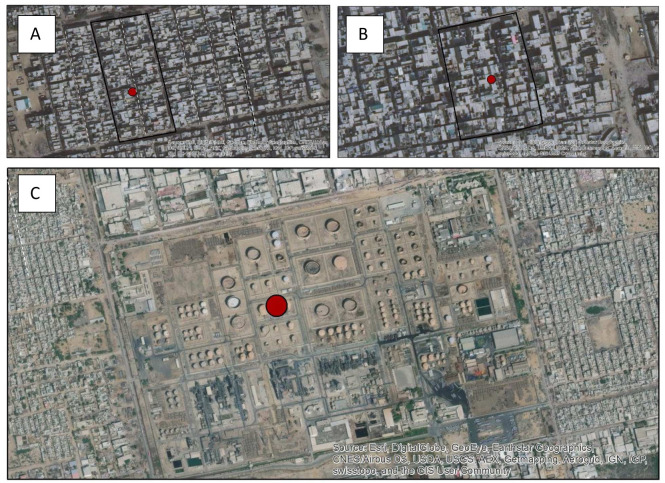
Satellite imagery of various parts of Karachi. Red dot depicts the random point. ( **A**) A residential area – with relevant organised layout. ( **B**) A block within a *katchi abadi* where buildings are more haphazardly spaced. ( **C**) An industrial area which would be excluded, and another random point selected.

**Figure 3.**
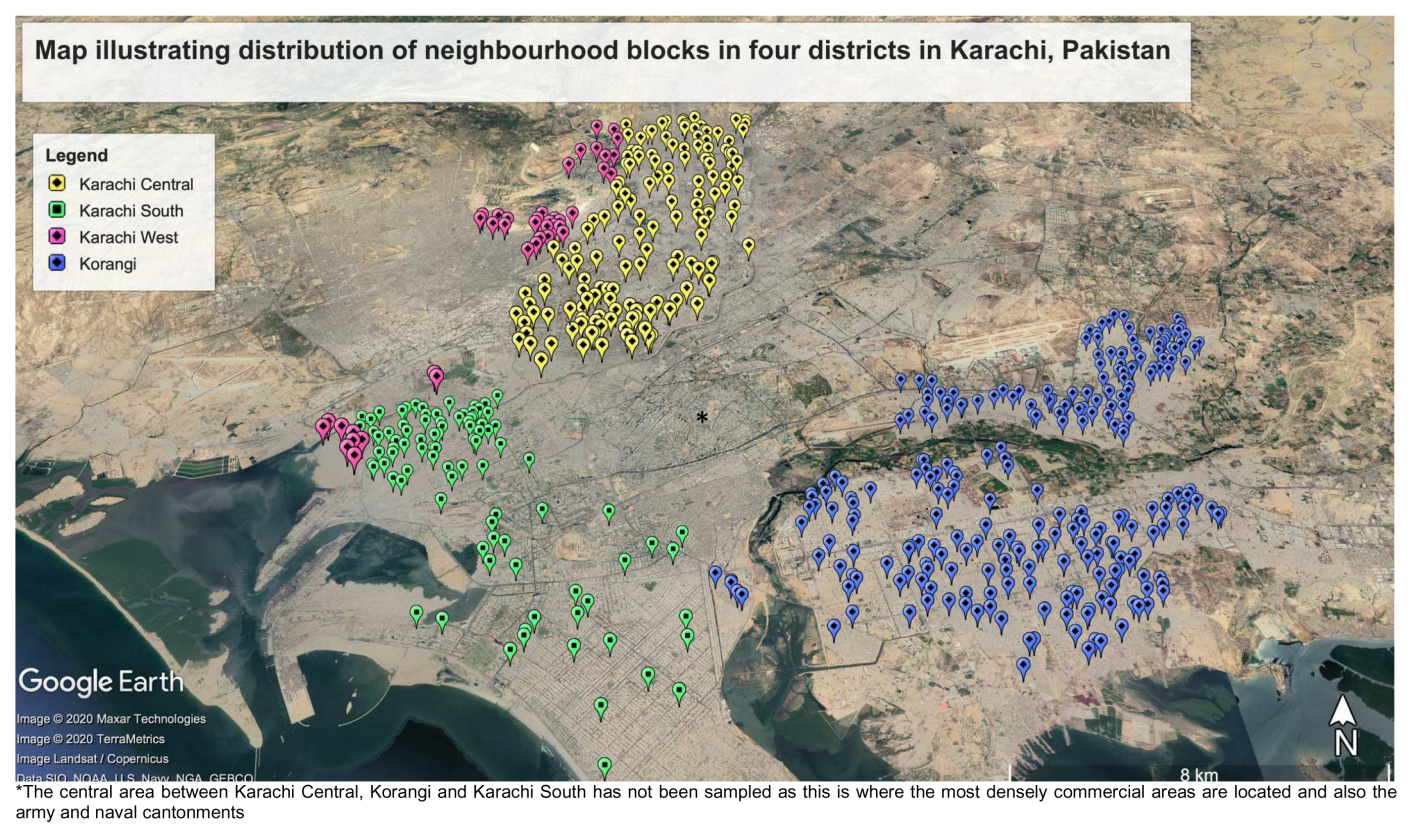
Map of Karachi showing distribution of neighbourhood blocks to be sampled by districts. *The central area between Karachi Central, Korangi and Karachi South has not been sampled as this is where the most densely commercial areas are located and also the army and naval cantonments.

*Selection of neighbourhood blocks*: ArcGIS ^®^ software (Environmental Systems Research Institute (ESRI), Release 10.5, Redlands, CA) is used to select random points within subdivisions of districts using the Select Random Points function. The random locations are then overlaid on the satellite imagery available through ArcGIS ^®^. If the point lies in a non-residential area it is excluded and the next point in the list is used. If the point is thought to lie in a residential area, a neighbourhood block (based on geographically adjacent households with approximately 200 individuals aged ≥ 15 years) is manually drawn on the map with the aim of keeping the random point in the middle of the block. The neighbourhood block is constructed using our prior experience of population sizes of differing blocks based on a qualitative assessment of the satellite imagery of the building structures in Karachi (for example, larger area outlined for areas where residential buildings are more spaced and smaller blocks for buildings closer together – see Figure 2). The random points, and outline of the block are then converted into KML format (file format used to display geographic data in Google Earth) and sent to the field teams, who can access them on smartphones to navigate to their defined blocks. Figure 3 shows the distribution of neighbourhood blocks sampled across the four districts of Karachi. 

### Study procedures

***Adult pulmonary tuberculosis prevalence survey.*** The procedures are based on the WHO methodology for tuberculosis prevalence surveys ^12^.

## Community sensitisation and enumeration

In brief, field workers conduct community sensitisation and enumeration of households with door-to-door census in each randomly selected neighbourhood block during weekdays (Monday to Thursday). If the neighbourhood block is non-residential or access is restricted for security reasons, e.g. naval or army residential areas and/or self-appointed community leaders (e.g. imam of the local mosque or community elders) do not endorse the survey, the block is deemed non-eligible. In that case the team moves into the next randomly selected block on the list and the reason for non-eligibility of the previous block is logged.

At the start of the community sensitisation, a local community head is identified and asked to lead community mobilisation and assist in the completion of a short socio-economic questionnaire at the neighbourhood block-level, including details on access to local healthcare facilities, schools, and water source. Table 2 outlines the definition of the survey terms used during enumeration.

**Table 2.** Definitions of survey terms.

Survey term	Definition
Neighbourhood block	A geographically localized community defined by the randomly selected point which is then outlined on the designated map (cluster size of approximately 200 adults)
Residential building	A building including a house, unit or flat where people live and sleep
Household	Basic residential unit defined by all members who share the main meal (usually evening)
Resident	Any person who has slept in the household the night before the start of enumeration

Enumeration of the households within a neighbourhood block starts with identification of all residential buildings in the block with the exclusion of empty plots, commercial buildings and schools. The team leader assigns a starting point of the block before enumeration begins. This is usually the building on the edge of the neighbourhood block closest to the main road. The team then move from residential building to the next nearest residential building, enumerating the households until a target of 200 adults per neighbourhood block is reached. 

## Field procedures

There are six field teams and each field team consisted of one team leader, two female counsellors and three male field workers during the week and then an additional mobile x-ray van driver, radiographer and phlebotomist at the weekends (Friday/Saturday/Sunday). Screening takes place at the weekend to maximise the chances of finding individuals in their homes, especially males. Households are visited a maximum of three times on different days (Friday/Saturday/Sunday) aiming to interview all adult household members and reduce the numbers of eligible participants being “missed”.

Eligible individuals (aged ≥ 15 years) who have given informed verbal consent undergo a questionnaire including information on previous and current tuberculosis treatment, tuberculosis symptoms (cough, haemoptysis, fever, night sweats and weight loss), history of smoking and diabetes. They are all referred to the x-ray van stationed in the neighbourhood for a digital CXR. A computer-aided software for reading radiological signs of tuberculosis, CAD4TB™ (Diagnostic Image Analysis Group, Radboud University Medical Center, Nijmegen, the Netherlands) is used to evaluate CXRs in this survey. The software identifies shape and textural abnormalities in chest x-ray images to produce an abnormality score ranging from 0 (normal) to 100 (highly abnormal) ^13^. A cut off CAD4TB score of 65 is used in the survey to identify an ‘abnormal’ CXR based on analysis of local programmatic data examining the association of CAD4TB score and prevalence of Xpert-positivity ^14^.

If either the tuberculosis symptom screen is positive (cough duration ≥ 2 weeks and/or fever and/or weight loss and/or night sweats and/or haemoptysis) or CAD4TB score ≥ 65, individuals are asked to submit two sputum samples, an instructed spot sputum sample on the same day and an early morning sputum. Individuals who are already on tuberculosis treatment at the time of screening are asked to submit two (on the spot and early morning) sputum samples, irrespective of symptom screen and digital CXR. Individuals with physical disabilities that prevent them from being able to mobilise from their home to the mobile x-ray van and pregnant women undergo a symptom screen only and are asked to submit two sputum samples irrespective of symptoms. Contact numbers of all individuals who submit a sputum sample are recorded. If the Xpert MTB/RIF Ultra (Cepheid, Sunnyvale, CA, USA) result is ‘trace’-positive, an additional sample (third sample) is collected, if possible, for repeat Xpert MTB/RIF Ultra testing. All sputum samples are collected by the field workers and transported to the Indus Hospital central laboratory for processing.

All study participants who are Xpert MTB/RIF Ultra positive including ‘trace positive’ on any samples are referred to the nearest health facility for clinical review and to start tuberculosis treatment if appropriate. We plan to contact all participants who are referred for either tuberculosis treatment or further clinical review within one month of referral to ascertain outcome, e.g. start of tuberculosis treatment or need for further clinical observation etc. Study participants found to have respiratory symptoms, such as chronic cough or difficulty breathing are also referred to attend the nearest health facility for further examination irrespective of Xpert MTB/RIF Ultra test.

### Child *M. tuberculosis* infection survey

Children aged 2 to 4 years old are recruited from the same households as the adult participants for the tuberculosis prevalence survey. Guardians of children aged 2 to 4 years old are asked for informed verbal consent (see *Ethics* section below), and complete a brief verbal questionnaire including a symptom screen (cough, fever, weight loss, failure to thrive and decreased playfulness), history of tuberculosis treatment, and history of any tuberculosis contact within last two years. 

A phlebotomist trained in paediatric venepuncture collects 4ml of blood from all children whose guardians’ consent to blood collection for QuantiFERON-TB ^®^ Gold Plus (QFT ^®^-Plus) according to manufacturer’s instructions. Blood samples are transported to the Indus Hospital central laboratory in Korangi within 6 hours of venepuncture. Children with positive QFT results are referred to the closest of one of three hospitals, which provide specialist childhood tuberculosis care, where the child is assessed and evaluated for tuberculosis, including a full diagnostic work-up by a trained paediatrician. Once tuberculosis has been excluded, children are started on isoniazid preventive therapy and followed-up for duration of treatment by the childhood tuberculosis programme.

**Figure 4.**
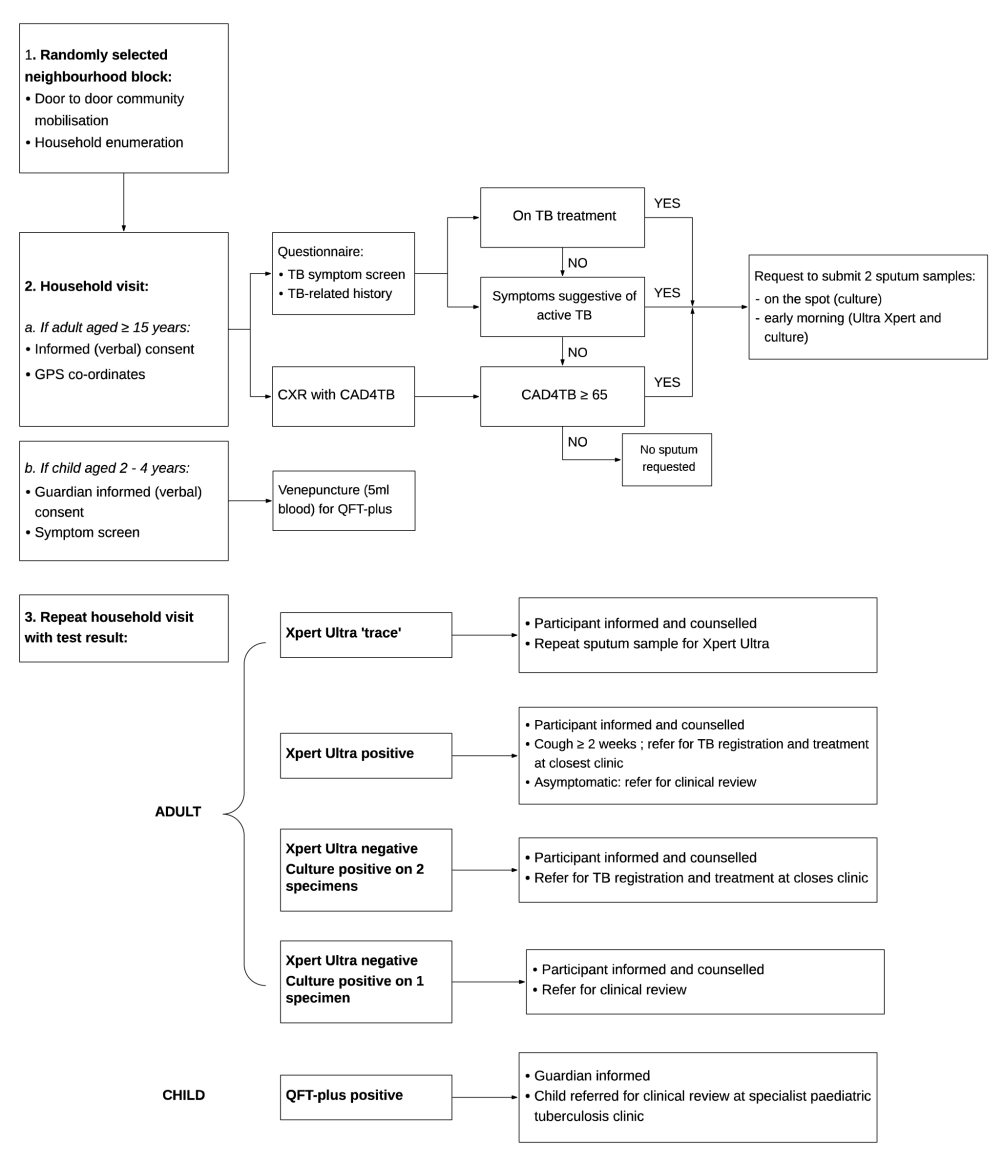
Flowchart of study procedures.

Figure 4 summarises the study procedures for the adult and child surveys.

## Laboratory procedures

### Adult pulmonary tuberculosis prevalence survey

Sputum samples transported in cooler boxes on the day the samples are submitted and are decontaminated using the standard NAOH-NALC method (final NAOH concentration 1% prepared inhouse). The pellet is re-suspended in 1.5 ml of normal saline. 0.8 ml, 0.5ml and 0.2ml of the suspension of early morning samples or on-spot samples (if no early morning sample collected) are tested using Xpert MTB/RIF Ultra, liquid culture (BACTEC™ Mycobacterial Growth Indicator Tube (MGIT™) 960 [BD])) and solid media (Lowenstein Jensen (LJ) [BD]) respectively. If both an on-spot and early morning sample is available, the early morning sample is inoculated in liquid and solid culture only. Cultures are incubated at 37°C in the automated MGIT960 and standard incubator system for a maximum of 42 and 56 days. Growth is identified by immune chromatographic identification test (Standard Diagnostics, Korea) for *M. tuberculosis*.

Laboratory cross-contamination is investigated as per local laboratory standard operating protocol in the event where two samples processed on the same day grow *M. tuberculosis* or when a single culture-positive sputum result (Xpert Ultra-negative and culture-negative on the other sample) is obtained for a study participant.

### Child *M. tuberculosis* infection survey

The blood samples are incubated at 37 ± 1°C for 16–24 hours in the Indus Laboratory within 16 hours after collection. After incubation, the tubes are centrifuged for 15 minutes at 2000–3000 x *g* and the plasma harvested. The standard QFT Plus ^®^ enzyme-linked immunosorbent assay (ELISA) kit is used. Samples from 22 subjects are analysed on a single plate in each run of ELISA. The optical density of each well is measured on a plate reader (Diatek DR-200 Bs microplate reader) using the QFT-Plus analysis software (Ascent Software Version 2.6, Thermo Scientific). The concentration of released interferon-gamma (IFN-γ) in each tube is calculated by subtracting the value of the nil (negative control) tube. If the coefficient of variation for the result is less than 15% and the correlation coefficient for the standard curve is greater than 0.98, the assay is technically valid. All the results are interpreted by referring to a 4-point standard curve as per the manufacturer’s instructions.

## Case definitions

*1. Adult pulmonary tuberculosis prevalence survey:*

**Definite bacteriologically confirmed pulmonary tuberculosis case** is defined as study participant with one culture-positive sputum specimen **and** at least one of the following conditions:
  - Xpert Ultra-positive
  - Culture-positive (either LJ or MGIT) in another specimen
**Probable bacteriologically confirmed pulmonary tuberculosis case** is defined as a study participant with an Xpert Ultra-positive sputum specimen but culture-negative on one or both sputum samples or a study participant with a single culture-positive sputum where laboratory cross-contamination has been excluded.
  These definitions deviate from the case definitions recommended by the WHO Global Task Force on TB impact measurement for tuberculosis prevalence surveys ^15^. Firstly smear-status has not been included as part of the case definition as smear-microscopy has been replaced by use of Xpert Ultra in this setting. Abnormal CXRs are also not included as part of the case definition as only computer-assisted detection of lung abnormalities are undertaken as part of the survey as a triage tool for sputum submission. Due to budget limitations, there is no central audited reading of CXRs by a trained radiologist and therefore CXRs are only used to identify individuals eligible for sputum submission rather than as a diagnostic modality of pulmonary tuberculosis. If a participant has no symptoms and the CAD4TB score <65 then Xpert Ultra and culture results were assumed to be negative.

*2. Child M.tuberculosis infection survey:*

***M. tuberculosis* infection** is defined as having a positive QuantiFERON ^®^-TB Gold Plus (QFT ^®^-Plus) assay. A QFT ^®^-Plus assay is considered positive, as per the definition recommended by the manufacturer, if either TB Antigen tube interferon-gamma (IFN-γ) response is significantly above the Nil IFN-γ value (≥0.35 IU/ml and ≥25% of the Nil IFN-γ value). For the test to be valid, the positive control (Mitogen) minus the Nil IFN-γ value must be ≥0.5 IU/ml and the Nil IFN-γ/ml value must be ≤ 8.0 IU/ml.

## Data collection and sources

The data collection tool is a custom-built application using an Android tablet linked to the OpenMRS platform.

The application includes: (i) an enumeration form at the neighbourhood block level which collects data including number of buildings and type (e.g. residential or commercial or institutional), and number of residential buildings accessed in each neighbourhood block, (ii) an enumeration form at the building level which collects data on the number of dwellings (e.g. separate apartments) within each building, number of households within each dwelling, total number of males and females in each household and the numbers of male and female eligible study participants (aged 15 years and above and between 2 to 4 years of age); (iii) individual-level questionnaire (separate for adults and children) which collects data on symptoms, history of tuberculosis and tuberculosis contact, and history of smoking and diabetes for adults only; (iv) a CXR form (adult only survey) which collects data on whether CXR was performed, refused or missed, and if performed, records the CAD4TB score; and (v) a specimen collection form for sputum sample and an IGRA test, which records whether the test was performed, refused or missed. If a specimen is collected the field staff complete the specimen collection form, the application automatically generates a unique order form identifier for that sample which then serves as the specimen number. The laboratory results of all tests conducted are entered into the associated OpenMRS software installed in the laboratory using the unique study identifier and specimen number which allows linkage of the field and laboratory data within the OpenMRS database.

Each field team also keeps a field log which includes a neighbourhood block identifier, building, dwelling and household identifiers, unique study participant identifier including basic demographic information and mobile contact numbers, CXR scores, whether a specimen was collected (sputum or blood). Due to limited resources a household level socioeconomic questionnaire is not administered; however, a neighbourhood block level paper-based questionnaire is completed at the community mobilisation stage to gauge the socioeconomic status of the neighbourhood. Study staff undergo systematic training on how to collect all data including data pertaining to symptom screen, CXR and specimen collection before the start of the study with refresher training every 4 months.

Once informed verbal consent has been obtained, a unique identifier is assigned to each study participant and all study participants are given a paper enrolment card with their allocated unique study identifier, which the study participant then presents at the x-ray van or any subsequent contact with the study team. Sticker labels of each unique study identifier are available in the form of a Quick Response (QR) barcode (machine-readable optical label) which are applied to the study enrolment card to minimise manual data entry of study identifier. All sputum samples collected are also labelled with unique study identifier QR barcode.

Data are stored in a data warehouse and downloaded on a daily basis. These data are cleaned and validated on a weekly basis before being merged into the final database by the data management team.

### Data confidentiality

Data confidentiality is strictly maintained. A log for each neighbourhood block is kept in a paper register which includes the names and contact details of study participants linked to their study identifier. These logs are kept locked in a cabinet in the office which is only accessible to the project manager. Questionnaires are identified using the unique identifier. All forms use a format that has been used by IRD for the last 10 years, which have a built-in suitability for ensuring consistent data collection using hand-held mobile devices. Data are stored in password-protected files accessible to a limited number of designated individuals. Data are secured by daily backing up of databases from the server. All research staff have Good Clinical Practice training and undergo refresher training courses in confidentiality and protocols are in place for the handling of data within the field team and in the data office.

## Efforts to address potential sources of bias

**Figure 5.**
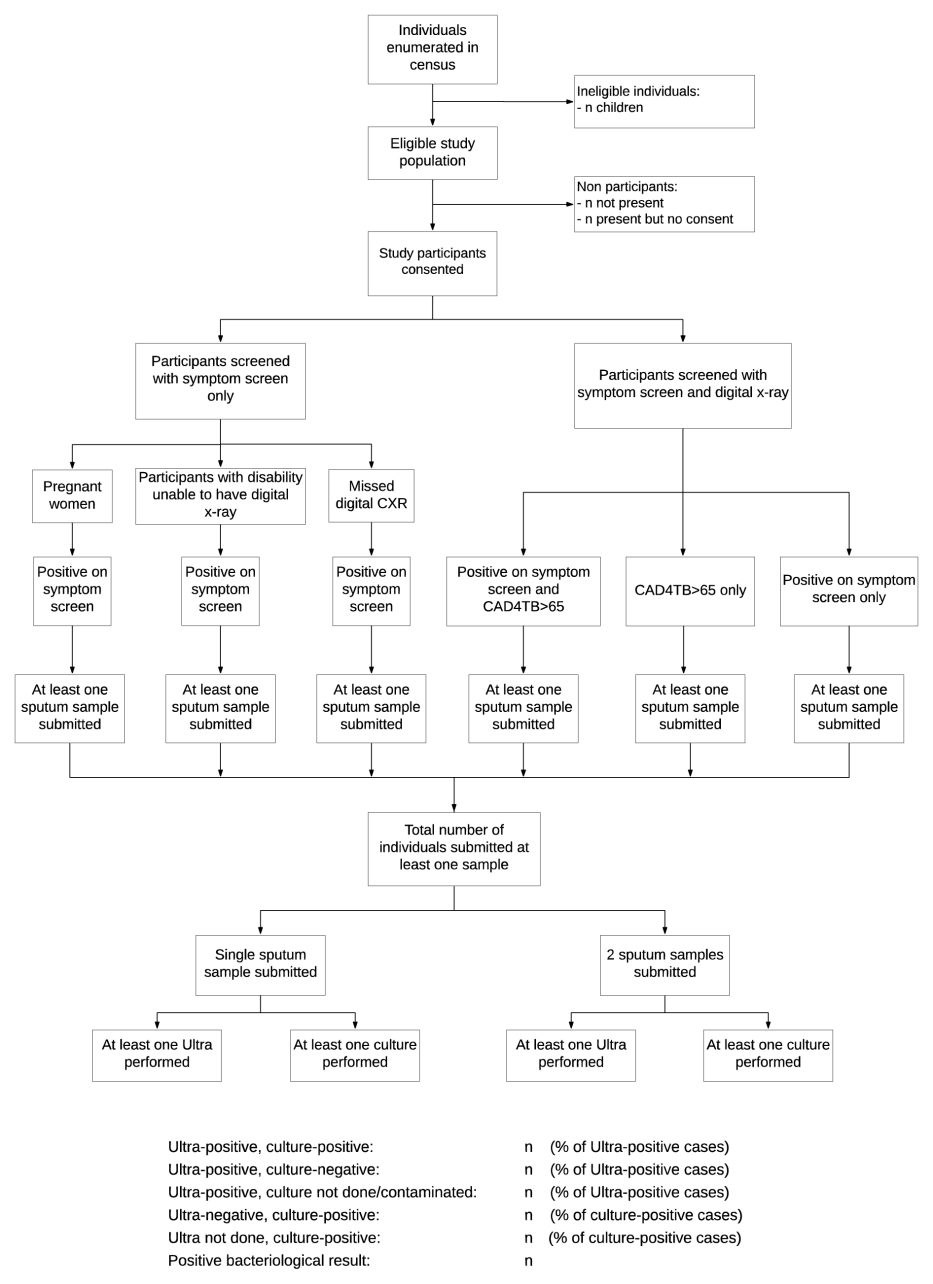
Schematic diagram of numbers of adult participants screened for tuberculosis according to the survey protocol.

The main potential source of bias is selection bias; however, enumeration of all eligible individuals and recording whether they were (i) a survey participant, (ii) absent or (iii) refused consent to participate allows an assessment for the presence of any systematic biases in the sampled population. The flow from eligibility through to participation and results will be presented in a flowchart as per the WHO Handbook for TB prevalence surveys (see Figure 5) ^15^. Biases for participation in the child IGRA survey can also be examined for whether those that participated are at a higher risk of *M. tuberculosis* infection using household-level data of known tuberculosis contact.

Another potential source of bias in the survey is missing data ^16^. A prevalence estimate that uses only individuals with complete data on pulmonary tuberculosis will be biased except under the strong assumption that those with full information are a random subset of eligible study population, which is unlikely to be the case. Methods that incorporate multiple imputation as part of individual-level analysis are less biased under a weaker assumption, and thus imputation will be used to obtain a more valid estimate of tuberculosis prevalence and to assess the bias of simpler approaches, such as complete case and cluster-level analyses. Addressing this source of bias is addressed in the section below as part of the statistical analysis.

## Statistical methods

### Adult pulmonary tuberculosis prevalence survey

***Completeness of data and internal consistency of data.*** Due to the interview process in the field all those who have given verbal consent have a symptom screen. Data on the number of randomly chosen neighbourhood blocks which declined participation or where deemed inaccessible by the survey team will be presented. A flowchart (see Figure 5) will be completed for all neighbourhood blocks included which will provide a description of the flow from enumeration through to participation in the different stages of the survey ^16^. In addition, an overall comparison of age and sex distribution between the Karachi population as per the 2017 census with the demographic distribution of the survey population will be undertaken. 

***Estimation of pulmonary tuberculosis prevalence (primary outcome).*** Cluster-level (cluster defined as neighbourhood blocks), and individual-level analyses taking into account clustering, will be undertaken to estimate the prevalence of bacteriologically confirmed pulmonary tuberculosis.

## Complete case analysis using cluster-level data

As Korangi district was over-sampled, a weighted analysis will be undertaken. For a cluster-level analysis, culture-confirmed tuberculosis prevalence among survey participants will be calculated separately for each cluster, and the average cluster-level prevalence will be calculated. An approximate 95% confidence interval will be calculated for the mean prevalence across clusters.

## Individual-level analyses

Individual-level analyses will be undertaken to enable adjustment for differences between participants and non-participants and multiple imputation of missing data (due to non-participation or lack of CAD4TB score or laboratory data), while simultaneously allowing for clustering in the sampling design ^16^. Three analyses, recommended by the WHO Global Task Force on TB impact measurement for analysis of tuberculosis prevalence surveys, will be undertaken to estimate the overall pulmonary TB prevalence ^15, 16^. One method does not take into account missing data and two methods correct for bias of missing data, including for survey non-participants and among participants missing data for CAD4TB and Xpert Ultra and/or culture results ^16^. The three approaches, all use logistic regression taking into account the clustered design, and are described below:

## Model 1. Robust standard errors (complete case analysis)

This model is restricted to survey participants and does not account for variation in the number of individuals per cluster, when estimating the point prevalence of pulmonary tuberculosis. Equal weight is given to each individual in the sample. It also excludes individuals who were eligible for sputum examination but for whom Xpert Ultra and/or culture results are missing. This method thus underestimates true prevalence amongst participants because data are missing for individuals who were eligible for sputum examination, who have a relative higher probability of being diagnosed with TB compared to those not eligible ^16^.

## Model 2. Robust standard errors with missing value imputation

This model uses (multiple) missing value imputation for individuals: a) without a CAD4TB score (including those who did not participate in the survey) and b) for individuals with a CAD4TB score ≥65 or tuberculosis symptoms but without an Xpert Ultra and/or culture results, in order to include all individuals who were eligible for the survey in the analysis. Variables to be considered in the imputation models where appropriate will include age, sex, symptoms, CAD4TB, Xpert Ultra and culture results, whether it is a *katchi abaadi*, zone and cluster. Cluster will only be used in imputation models with a common outcome. This model allows for both the clustering in the survey design and the uncertainty introduced by imputation of missing values when estimating the 95% confidence interval for the prevalence of pulmonary tuberculosis.

## Model 3. Robust standard errors with missing value imputation (for individuals eligible for sputum examination), and inverse probability weighting (applied to all survey participants)

Missing value imputation will be used for individuals eligible for sputum examination (defined as having a CAD4TB score ≥ 65 and/or tuberculosis symptoms) for whom data on CAD4TB score, Xpert Ultra and culture results are not available and inverse probability weighting applied to all survey participants. Survey participants are defined for this analysis as individuals who participated in the survey and were screened with at least a symptom screen. Inverse probability weighting is then used to correct for differentials in participation in the survey by age, sex, and cluster. Through the combination of imputation of missing data and the use of weights, the analysis aims to represent the whole of the survey eligible population (N _1_), but the weights are applied only to individuals who were screened with at least symptoms.

Comparisons of results across models will allow an assessment of the robustness of the estimates derived using different analytical approaches and model assumptions. A sensitivity analysis using the ‘probable TB case’ definition will also be undertaken.

### Child *M. tuberculosis* infection survey

The annual risk of infection (ARTI) will be estimated from the prevalence of *M. tuberculosis,* which ** is inferred from the number of positive QFT tests divided by the total number of QFT tests performed, using the standard R formula:

$$\text{R}=1-{\left(1-\text{P}\right)}^{1/\text{a}}$$

where R is the probability of being infected in any one year, P is prevalence of *M. tuberculosis* infection and a is the mean age at which the observed prevalence is estimated ^6^.

All analyses will be performed in Stata version 16 (Stata Corporation, College Station, USA) and R (The R Foundation for Statistical Computing, Vienna, Austria).

### Ethics and dissemination

The study protocol has been approved by the ethical review committee of Interactive Research Development (IRD)/The Indus Hospital Research Centre in Karachi in Pakistan (IRD reference number: IRD_IRB_2017_04_002) and the London School of Hygiene & Tropical Medicine (LSHTM) Observational/Interventions Research Ethics Committee (LSHTM ethics reference number: 12063).

Informed verbal consent is obtained from each household head before accessing the household. Verbal informed consent is then obtained from each eligible participant aged 15 years and above before enrolment into the study and after a thorough explanation of the risks and benefits of participating in the study. We specifically applied for a waiver with respect to obtaining informed verbal consent rather than informed written consent when applying for ethical approval from both ethics review committees because it would result in non-representative sampling in this setting where a large proportion of the population are illiterate and are reluctant to provide fingerprints due to concerns about personal security. The participant information is read to all participants in their preferred language (Urdu, Sindhi, and English). Any questions raised by the potential participants are answered before participation in the study. The voluntary nature of participation in the survey and the option to withdraw from the study at any time without affecting participant rights and benefits are explained. Consent for minors aged 2 to 4 years is sought from a parent or guardian. Radiation safety procedures are applied including protection and monitoring of the workers and participants. Pregnant women will not be x-rayed but will only have a symptom screen and asked to submit sputum samples irrespective of symptom status. To ensure maximum participation and minimise discrimination all individuals with a physical disability who are unable to mobilise to the x-ray van will have a symptom screen and asked to submit sputum samples irrespective of symptom status. All tuberculosis cases and participants needing care are referred to the nearest health facility.

Findings will be presented locally to the appropriate channels (regional and national meetings of the Provincial and National TB programme) and through the continuing professional development programme (CPD) at the Indus Hospital. This will allow Pakistan public health practitioners and policy makers to incorporate any relevant findings directly into health policy if appropriate. International dissemination will be through academic publications and presentations at international meetings. Results will also be shared with participating communities and collaborators.

## Data availability

No data are associated with this article.
