# Supplementary material for: Concurrent adult pulmonary tuberculosis prevalence survey using digital radiography and Xpert MTB/RIF Ultra and child interferon-gamma release assay Mycobacterium tuberculosis infection survey in Karachi, Pakistan: a study protocol
Source: Wellcome Open Res. 2020 Jul 6;5:159. [Version 1] doi: 10.12688/wellcomeopenres.15963.1 (PMC7968470; doi:10.12688/wellcomeopenres.15963.1)
Supplement: Peer review history

# Peer review: Reviewer response for version 1

**DOI:** 10.21956/wellcomeopenres.17507.r42314

**Published:** 2021-03-12

**Authors:** Kassu Desta

**Affiliations:** 1 Department of Medical Laboratory Science, College of Health Sciences, Addis Ababa University, Addis Ababa, Ethiopia

I felt that, the protocol is so interesting and it will help to take intervention to reduce Tuberculosis in the nation. However, I am not sure that the protocol addresses both active Pulmonary TB and Latent TB equally as described or adults and children respectively.

 Under the intervention section in the hospital the researchers under zero Tb Karachi initiative, Ultraviolet Germicidal Irradiation light installation in selected wards is mentioned as one of the  intervention to reduce TB. However, how such intervention could reduce person to person transmission of TB is not clearly discussed. Whether a single intervention or bundle of interventions have impact is not clearly shown in the protocol.

 Another important concern is the intervention time to reduce PTB significantly in 3-4 years time, if possible, the researchers could consider if this time is useful.

Is the study design appropriate for the research question?

Yes

Is the rationale for, and objectives of, the study clearly described?

Yes

Are sufficient details of the methods provided to allow replication by others?

Yes

Are the datasets clearly presented in a useable and accessible format?

Yes

Reviewer Expertise:

General Medical Microbiology and Laboratory Sciences including TB, HCAI, AMR and Colonization study

I confirm that I have read this submission and believe that I have an appropriate level of expertise to confirm that it is of an acceptable scientific standard.

# Peer review: Reviewer response for version 1

**DOI:** 10.21956/wellcomeopenres.17507.r40270

**Published:** 2020-09-25

**Authors:** Irwin Law

**Affiliations:** 1 Global TB Programme, World Health Organization, Geneva, Switzerland

Thank you for allowing me to review your well written protocol. I believe that it is an important piece of work, and look forward to seeing the results with a view to improving the TB situation in Pakistan, and to providing further lessons globally.

 I have included some comments below for your consideration and for clarification:

**Introduction**

- It’s mentioned in in introduction that an objective is to measure the “prevalence of *infectious* pulmonary tuberculosis”. How do you confirm if it is infectious or not? This probably delves into the issue of subclinical TB, and the possibility of whether an *asymptomatic* person with culture positive TB is infectious or not.
- The title and description of the Zero TB initiative in the introduction mentions “Ultra” but there is no mention of culture which is your primary diagnostic for defining a TB case. May be some mention of it is required here?
- Percentage of adult population residing in the *katchi abadis *at district level is at 8-18% – can I confirm this means there are more than 80% who are children/adolescents? Will this skew prevalence survey results if this age structure is dissimilar to other parts of your sampling frame?

**Sampling**

- In Cambodia, it took nearly 10 years (2002-2010) to see a decline of 38% in smear-positive TB prevalence ^1^. Similarly, between their respective surveys, in Myanmar  (2009-2017/18) there was a decline of 51% in culture positive TB, and in Viet Nam there was an estimated 29% reduction in prevalence.[ii] ^,^ [iii] In the Philippines (2007-2016), there was no measured decline in culture confirmed TB.[iv] Therefore, do you think activities and policies undertaken in the 4 years between the surveys in Pakistan will see an appreciable decline of 33% in burden?
- How are clusters going to be selected in the repeat survey? Will they be the same as the first survey?
- Typically in highly urban areas, participation rates can be very low. Even 75% is quite ambitious. Was any amendment made to the cluster size if this was consistently lower than 75%?
- As you are only sampling clusters that are <2 hours from HQ, is there any risk of bias towards one population especially related to health facility accessibility (assuming more rural areas have less accessibility)? Do you expect someone 2+ hours away to be different in terms of risk of disease? What is the limitation of the vehicle driving more than 2+ hours – is it due to security?
- A major advantage of using a fixed cluster size of 200 people in conjunction with PPS (population proportional to size) methodology is that, at the analysis stage, it is possible to use a self-weighting approach without any need to apply population weights. Did you select clusters based on population proportional to size methodology? If not, how will this be addressed during the analytical phase?
- If a cluster/block is rejected, will it be replaced by another? If so, how?

**Eligibility of participants**

- What is the eligibility criteria for someone to participate in the survey? Aside from age, should residency be included and explicitly mentioned as well e.g. slept in the household the previous night before enumeration.
- What is your denominator for determining your participation rate?
- Typically, an eligible population in a prevalence survey includes people who are eligible but do not want to participate or not present in that household. Are there any potential biases with their exclusion? e.g. men who work commonly do not participate - could they have a higher risk of TB?
- Is the cluster size based on 200 people per block who are enumerated or invited to participate in the survey? Ideally it should be the latter.

 
**Screening of participants**

- It appears that all eligible people are interviewed at home and not at a centrally located survey site in the cluster? If so, is there much of a drop off in the number of interviewed participants without a CXR (therefore participation may be lower overall)?
- As part of your tuberculosis symptom screen in adults, does fever, weight loss, night sweats and haemoptysis have a specified duration like cough?
- How was a CAD4TB cutoff of 65 determined? It will be interesting to see how many culture and Xpert Ultra positives you get with a CAD<65 and are symptom screen positive.
- How do you know if a woman is pregnant? Should it not be that all women (and men) are offered lead aprons prior to CXR screening?
- Although rates of HIV are low in Pakistan, was HIV testing of participants considered?

 
**Case management**

- In figure 4, what does “asymptomatic” mean? Or is it a person who is symptom screen negative (SSN) e.g. a person with only a cough <2 weeks would be classified SSN.
- In figure 4, you state that a person is a person with 2 culture positives specimens is referred for TB treatment but not if 1 specimen is culture positive. Even if the latter had symptoms would you not refer for treatment (assuming there is no cross contamination of culture in the lab)? However, if you have one Xpert positive with symptoms, you would refer for TB treatment. Can you explain the rationale for this?

 
**Data management**

- In light of budgetary limitations, for data management purposes (and subclinical TB evaluation), it would be useful to check all chest X-rays (CXR) of those with any positive laboratory result. There may be times, where you have a “normal” CXR, but the person has a positive Xpert Ultra and/or culture. This could then be due to data management error, lab error or the truth in which case this makes for interesting findings. Given the very few expected number of survey cases, validating the numerator is quite critical.

 
**Use of Xpert MTB/RIF Ultra**

- What was the rationale to collect a third sputum sample if Ultra was trace, and are two traces better than one i.e. are they truly independent results if they came from the same person? How will you define a participant who is trace positive and culture-positive?
- What is your thinking behind a past history of TB and a positive Xpert Ultra result (including trace)? If a *probable case* (i.e. Xpert Ultra only) has been treated for TB in the past year or month, and are still Xpert Ultra positive, but culture negative, then can you reasonably assume the person is bacteriologically negative? In recent surveys which used Xpert Ultra, we were being ultra (!) conservative and used CXR to define a case of *active* TB if Xpert Ultra was positive, culture was negative *and* they had no history of TB. This accounted for about 5-15% of total survey cases.
- What is the national policy for the use of Xpert Ultra in Pakistan? Are you assuming Ultra results obtained from the participants in the community are the same as Ultra results from patients screened in the clinical hospital setting?
- In Fig 5, is a trace call classified as Xpert Ultra positive?

 
**Analysis**

- In the analysis phase, it may be worth exploring the use of poststrata adjustment in case the enumerated census is different from the national census. An example is shown here: https://stats.idre.ucla.edu/stata/faq/how-do-i-analyze-survey-data-with-poststratification/
- Sometimes participants can submit samples but there is insufficient sputum volume or quality to perform culture, data management error, labeling error, lab transport delays etc and therefore labs may reject the sample. Therefore, available results may be another useful process indicator e.g. Proportion of those eligible to submit with a valid culture result i.e. MTB positive, negative, MOTT (and not missing, or contaminated).
- It will be interesting to see the prevalence:notification ratio in your survey by gender. Afghanistan and Pakistan have for a long time always notified more cases of TB in women than in men. Globally, other countries have more men than women being notified.

**Very minor comments:**

- Under study rationale, you state Xpert MTB/RIF but may be better to state Xpert MTB/RIF Ultra so as not to confuse between generations of the test.
- X-ray has a capital X
- Non-governmental has no hyphen and is one word 

Thank you.

Is the study design appropriate for the research question?

Yes

Is the rationale for, and objectives of, the study clearly described?

Yes

Are sufficient details of the methods provided to allow replication by others?

Partly

Are the datasets clearly presented in a useable and accessible format?

Not applicable

Reviewer Expertise:

National TB prevalence surveys

I confirm that I have read this submission and believe that I have an appropriate level of expertise to confirm that it is of an acceptable scientific standard.

## References

[1] : Cross-sectional studies of tuberculosis prevalence in Cambodia between 2002 and 2011. *Bull World Health Organ*.2014;92(8) : 10.2471/BLT.13.131581 573-81 10.2471/BLT.13.131581 25177072PMC4147411

[2] : WHO monitoring mission report of Viet Nam’s second prevalence survey.2019;

[3] 4th National TB Prevalence Survey 2017/2018. *Myanmar Short Report*.2019;

[4] National TB prevalence survey. *Republic of the Philippines*.2016; Reference source
